# Test–re-test reliability and inter-rater reliability of a digital pelvic inclinometer in young, healthy males and females

**DOI:** 10.7717/peerj.1881

**Published:** 2016-03-31

**Authors:** Chris Beardsley, Tim Egerton, Brendon Skinner

**Affiliations:** 1 Strength and Conditioning Research Limited, London, United Kingdom; 2 Sport Science Tutor, Congleton, United Kingdom; 3 Department of Sports Therapy, Staffordshire University, United Kingdom

**Keywords:** Reliability, Pelvic tilt

## Abstract

**Objective.** The purpose of this study was to investigate the reliability of a digital pelvic inclinometer (DPI) for measuring sagittal plane pelvic tilt in 18 young, healthy males and females.

**Method.** The inter-rater reliability and test–re-test reliabilities of the DPI for measuring pelvic tilt in standing on both the right and left sides of the pelvis were measured by two raters carrying out two rating sessions of the same subjects, three weeks apart.

**Results.** For measuring pelvic tilt, inter-rater reliability was designated as good on both sides (ICC = 0.81–0.88), test–re-test reliability within a single rating session was designated as good on both sides (ICC = 0.88–0.95), and test–re-test reliability between two rating sessions was designated as moderate on the left side (ICC = 0.65) and good on the right side (ICC = 0.85).

**Conclusion.** Inter-rater reliability and test–re-test reliability within a single rating session of the DPI in measuring pelvic tilt were both good, while test–re-test reliability between rating sessions was moderate-to-good. Caution is required regarding the interpretation of the test–re-test reliability within a single rating session, as the raters were not blinded. Further research is required to establish validity.

## Introduction

Traditionally, pelvic tilt has often been measured in clinical practice to identify the presence of abnormal postures that may cause dysfunction and lead to chronic musculoskeletal pain conditions (Herrington, 2011), such as low back pain (Juhl, Cremin & Russell, 2004). However, in cross-sectional studies, anterior pelvic tilt has not often been identified as a risk factor for low back pain (Youdas et al., 2000; Chaléat-Valayer et al., 2011), although more recently Lim, Roh & Lee (2013) found that anterior pelvic tilt was lower in healthy individuals than in subjects with low back pain and Youdas et al. (2000) reported a significant correlation between the pelvic tilt angle and Oswestry Disability Index scores in females. Even so, Lim, Roh & Lee (2013) reported no similar, significant correlation when carrying out the same calculation. Such conflicting results may reflect the necessity for a biopsychosocial model to explain such conditions rather than a purely patho-anatomical one (O’Sullivan, 2012).

In addition, both in the literature and anecdotally, there are reports that greater anterior pelvic tilt may increase the risk of musculoskeletal injury during running (Schache et al., 1999; Schache, Blanch & Murphy, 2000; Schache et al., 2002). It has been suggested that such injuries could occur either through repetitive impingement of the vertebral facets (Schache et al., 1999; Schache et al., 2002) or by producing excessive lengthening of the hamstring, leading to strain injury (Schache et al., 1999; Schache et al., 2002). On this or another basis, some clinicians may decide to measure the extent of anterior pelvic tilt in their patients and clients, particularly those who undertake regular running activities.

Pelvic tilt can be measured either with a single measurement, at the center line, or with two measurements at either lateral border. Measurements taken in cadavers have shown that differences in bony anatomy lead to significant between-side differences in anterior pelvic tilt (Preece et al., 2008) and significant differences in pelvic tilt between sides have also been reported in live subjects (Herrington, 2011). The difference in pelvic tilt between sides has been taken as a measurement of pelvic torsion, which some investigations have associated with leg length discrepancy (Cummings, Scholz & Barnes, 1993; Young, Andrew & Cummings, 2000; Betsch et al., 2012; Wild et al., 2014). It has been variously suggested that pelvic torsion occurs as a natural adaptation to leg length discrepancy (Krawiec et al., 2003), that greater anterior pelvic tilt occurs on the side of the shorter leg compared to the contralateral leg (Knutson, 2005), and that this biomechanical feature may be common to both symptomatic and asymptomatic individuals alike (Herrington, 2011). Even so, the precise relationships between leg length discrepancy and pelvic torsion, as well as between leg length discrepancy and musculoskeletal injury risk, are contentious and remain poorly understood (Gurney, 2002; Juhl, Cremin & Russell, 2004; Knutson, 2005; Cooperstein & Lew, 2009).

Several methods are available for measuring pelvic tilt. Early studies often used radiography (Clayson et al., 1962; Flint, 1963) and this method continues to be used in relation to surgery affecting the hip and pelvis (Blondel et al., 2009; Lazennec et al., 2011) or when a standard is required against to validate other methods (Burdett, Brown & Fall, 1986; Crowell et al., 1994; Petrone et al., 2003; Sprigle et al., 2003; Lazennec et al., 2011). Other methods include the Iowa Anatomical Position System (Day, Schmidt & Lehmann, 1984), the Metrecom Skeletal Analysis System (Barakatt et al., 1996), the antenna method (Moes, 1998), goniometers (Burdett, Brown & Fall, 1986; Sprigle et al., 2003), calipers (Sanders & Stavrakas, 1981; Gajdosik et al., 1985; Alviso, Dong & Lentell, 1988), inclinometers (Walker et al., 1987; Heino, Godges & Carter, 1990; Crowell et al., 1994; Youdas et al., 1996; Levine, Walker & Tillman, 1997; Hagins et al., 1998; Petrone et al., 2003; Preece et al., 2008; Gnat et al., 2009; Herrington, 2011), low-dose digital stereoradiography (Lazennec et al., 2011; Guenoun et al., 2012), and magnetic resonance imaging (MRI) scans (Lalonde et al., 2006).

Calliper-based inclinometers seem to be among the most common tools used by clinicians for measuring pelvic tilt for several reasons. They display good reliability for measuring iliac crest height differences (Walker et al., 1987; Hagins et al., 1998; Petrone et al., 2003; Krawiec et al., 2003) and for measuring pelvic tilt (Heino, Godges & Carter, 1990; Crowell et al., 1994; Youdas et al., 1996; Gnat et al., 2009; Herrington, 2011; Fourchet et al., 2014). Using the intra-class correlation coefficient (ICC) to assess reliability, researchers investigating the use of calliper-based inclinometers in healthy adult volunteers have generally reported at least good (Walker et al., 1987; Heino, Godges & Carter, 1990; Herrington, 2011) if not excellent reliability (Youdas et al., 1996; Hagins et al., 1998; Krawiec et al., 2003; Gnat et al., 2009). Additionally, calliper-based inclinometers have also been found to display good convergent criterion reference validity by reference to radiography (Crowell et al., 1994; Petrone et al., 2003). Furthermore, these devices also have several practical advantages to the clinician, being quickly and easily utilized (Crowell et al., 1994), as well as being small, portable, relatively safe compared to radiography, and comparatively inexpensive in comparison with low-dose digital stereoradiography and MRI scanning devices. Calliper-based inclinometers also permit measurements to be taken on both sides of the pelvis, which may be important given the differences between sides that have previously been observed (Preece et al., 2008; Herrington, 2011).

**Figure 1:**
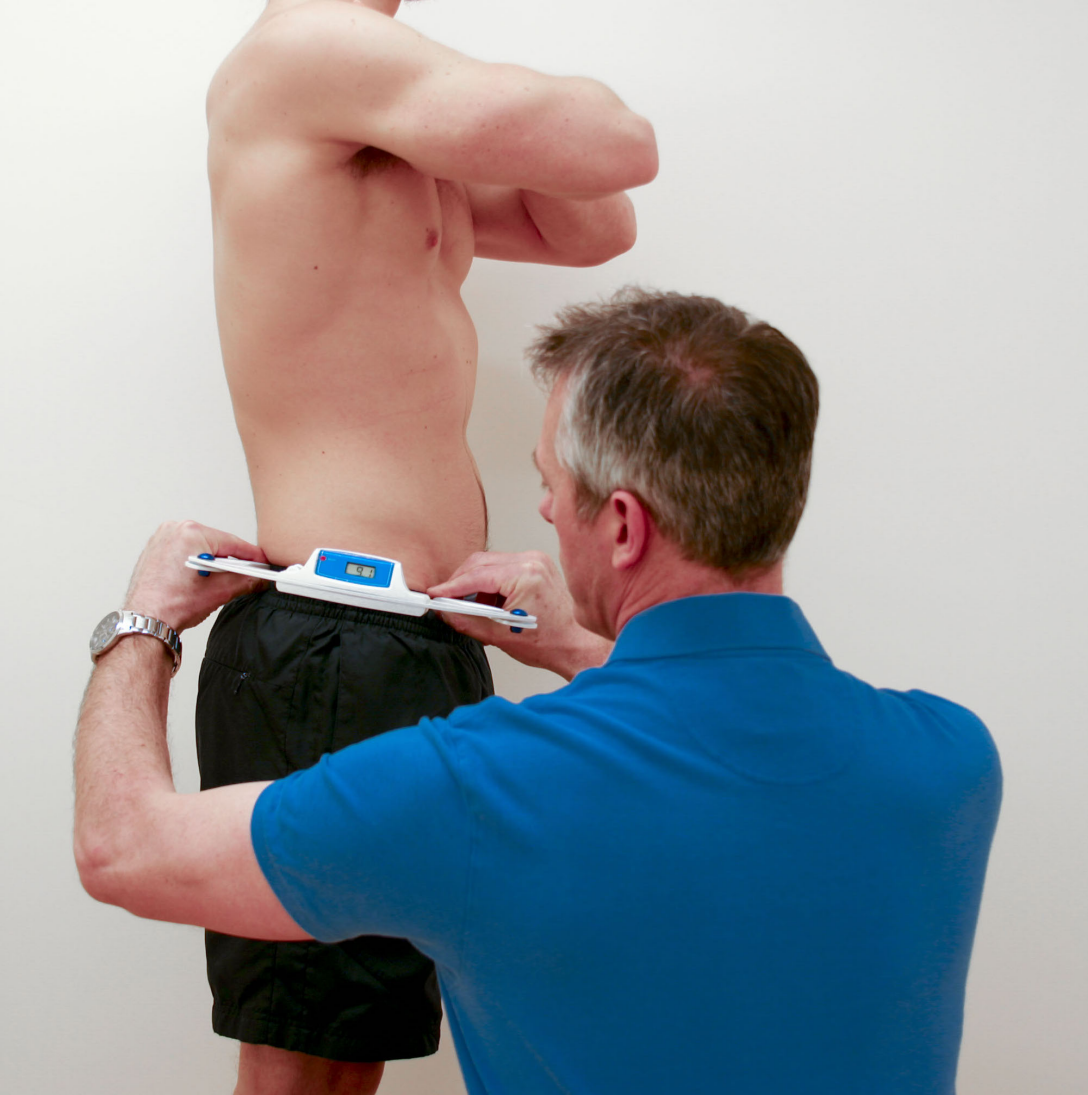
Measuring pelvic tilt using the DPI. Standing position maintained by subject, while rater measured pelvic tilt using the DPI.

Different models of calliper-based inclinometer have been investigated in the literature. The Palpation Meter (PALM, Performance Attainment Associates, St. Paul, MN, USA) is the calliper-based inclinometer that has been extensively explored (Hagins et al., 1998; Petrone et al., 2003; Krawiec et al., 2003; Gnat et al., 2009; Lee, Yoo & Gak, 2011; Herrington, 2011; Fourchet et al., 2014). Other models that have been investigated include those developed and modified by Walker et al. (1987) and Crowell et al. (1994). The model used and developed by Crowell et al. (1994) included a spirit level to permit readings relative to the ground, finger-tip rings to allow superior palpation of the bony prominences, and a digital read-out for ease and speed of reading the output. The Digital Pelvic Inclinometer (DPI, Sub-4 Limited, UK) is a new, commercially-available, calliper-based inclinometer that is very similar to the model developed by Crowell et al. (1994) (Fig. 1). Like the model developed by Crowell et al. (1994), the DPI uses a digital display. This display allows the clinician to see the output of the device while performing the measurement procedure. In addition, the DPI also has recessed calliper ends, which allow simultaneous palpation of the bony prominences with the hands and the calliper arms. Finally, the DPI also contains a spirit level to facilitate measurements of pelvic angles relative to the ground as well as relative to the other side of the pelvis.

The purpose of this study was to investigate the inter-rater reliability and test–re-test reliability of the DPI in young, healthy males and females across two rating sessions with experienced, trained raters. The first hypothesis for this study was that inter-rater reliability for the DPI between two raters would be good. The second hypothesis was that test-rest reliability for the DPI would be good by reference to three separate measurements taken on a single rating session. The third hypothesis was that test–re-test reliability for the DPI would be good by reference to the mean of the measurements taken on each of two rating sessions on separate occasions.

## Method

### Experimental approach

The inter-rater reliability and test–re-test reliabilities of the DPI for measuring pelvic tilt on both the right and left sides of the pelvis were measured by two raters carrying out two rating sessions of the same subjects on separate occasions. The dependent variables were the two angles of pelvic tilt (right and left sides). The independent variables were the test number (3 tests per session), the session (2 sessions), and the rater (2 raters).

### Measurement procedures

The subjects arrived at the laboratory wearing athletic clothing. The subjects were tested by both raters in two sessions on two separate days, three weeks apart. The two raters were in separate rooms (bays) and were therefore blinded from the results recorded by the other. Each rater was supervised by an investigator to ensure that no information could be passed from one to the other, and all data was retained by the investigators to prevent any communication of results between raters. In addition, the subjects were blinded from the results, and could not pass details between raters. The subjects were measured while standing in a normal, relaxed position and wearing loose clothing but no shoes or footwear on a level floor in the same room of the same building, at the same time of day on each occasion, as shown in Fig. 1. No specific instructions were provided to the subjects regarding posture in order that measurements during a normal standing position could be recorded. The raters used a DPI to take measurements for pelvic tilt on each side of the pelvis (right and left). The DPI is a hand-held, calliper-based inclinometer with a digital readout (Fig. 1).

The DPI comprises two precision arms, which are mounted upon a main body. The main body contains a tri-axial accelerometer, which records the angle of pelvic tilt across the two precision arms. The output from the tri-axial accelerometer is shown as an angle in degrees, in numerical form on a liquid crystal display. For each measurement of pelvic tilt, standard instructions were used per the manufacturer’s guidelines, as follows: “the practitioner places the index finger and thumb on each hand on each finger grip at the end of the DPI arms. With each index finger slightly prominent ready for concurrent palpation of the posterior superior iliac spine (PSIS) and anterior superior iliac spine (ASIS), the practitioner positions the DPI on the side of the innominate bone and takes a reading. The practitioner moves their index finger over the most prominent point of the iliac crests until the apex is established for the measuring. The practitioner then reads off the degree of inclination from the LCD.”

### Subjects and raters

Following a power analysis as described by Wolak, Fairbairn & Paulsen (2012), a convenience sample of 18 healthy subjects (12 males and 6 females) were recruited from a university physical therapy program. Of the 18 subjects, only 16 were included in the test–re-test reliability assessment between sessions (for subject characteristics relevant to each assessment, see Table 1).

**Table 1:** Descriptive statistics for the subjects.

	Inter-rater	Test–re-test (within sessions)	Test–re-test (between sessions)
Number of subjects	18	18	16
Number of males (m) and females (f)	12m/6f	12m/6f	11m/5f
Age (years)	23.6 ± 4.7	23.6 ± 4.7	24.0 ± 5.0
Bodyweight (kg)	74.7 ± 13.5	74.7 ± 13.5	76.2 ± 14.0
Height (m)	1.74 ± 0.08	1.74 ± 0.08	1.75 ± 0.09

Subjects qualified for the study if they met the following criteria: were ≥18 years of age, were able to stand unsupported for the duration of the measurement process (<10 min), were free from existing low back injuries, had not experienced any low back injuries within the previous 3 months, and had no medical condition leading to clinically meaningful leg length inequality. In accordance with ethical requirements, the subjects received an explanation of the nature, purpose, and risks of the study and were given the opportunity to ask questions. All subjects signed an informed consent document prior to participating in the study. Written ethical approval for the study was granted by the Faculty of Health Sciences Ethics Panel, Staffordshire University.

A convenience sample of two raters with similar experience in using the DPI were recruited. They completed the DPI measurements for all subjects. The first rater was a sports podiatrist with 26 years of experience in clinical practice, and 4 years of experience with using the DPI. The second rater was a podiatrist with 15 years of experience in clinical practice, and 6 months of experience with using the DPI.

### Statistics

Intra-class correlation coefficients (ICC) were used to assess the inter-rater, intra-rater (between sessions) and intra-rater (within sessions) reliability of pelvic tilt measured using the DPI for both right and left sides. ICCs are suitable for use in fully-crossed study designs assessing reliability of interval variables (Hallgren, 2012). Since the raters were not randomly selected for each subject but were the same for all subjects, a two-way Analysis of Variance (ANOVA) model was used (Shrout & Fleiss, 1979). Since absolute rather than ranked values of pelvic tilt are of interest, the ICC model type was set to require absolute agreement (McGraw & Wong, 1996). The unit of measurement used in the model differed between the statistics calculated. Since clinical practice commonly involves taking multiple measurements and recording the mean, the mean of the three ratings taken for each subject in a single session was used for hypothesis testing for inter-rater reliability and test–re-test reliability between sessions. Inter-rater reliability was assessed by combining the results of both testing sessions. Test–re-test reliability between sessions was assessed by combining the results of both raters. In contrast, for test–re-test reliability within single sessions, the reliability of the single, individual ratings was assessed, although again the results of both raters were combined together (Shrout & Fleiss, 1979). Before commencing the trial, it was decided that interpretation of the reported values for each ICC would be based upon the following criteria: <0.50 = poor, 0.50–0.75 = moderate, and >0.75 = good (Walmsley & Amell, 1996; Batterham & George, 2003; Portney & Watkins, 2008). To enhance clinical interpretation of the results, the standard error of measurement (SEM) and minimum difference to be considered real (MD) were estimated (Weir, 2005). Descriptive statistics were calculated as means with standard deviation. Statistical significance was set a priori at *p* < 0.05. All statistical analysis was performed using R, using the irr (Gamer et al., 2007) and ICC (Wolak, 2012) packages.

## Results

### Descriptive statistics

Descriptive statistics (mean ± standard deviation) for pelvic tilt on the right and left sides are presented in Table 2.

**Table 2:** Descriptive statistics for pelvic tilt.

	Right (degrees)	Left (degrees)	Difference (degrees)
Mean	10.6	10.5	0.1
Standard deviation	5.0	5.8	3.8

### Reliability

The ICC, SEM, and MD reported when measuring inter-rater reliability, test–re-test reliability (within sessions) and test–re-test reliability (between sessions) are presented in Table 3.

**Table 3:** Inter-rater and test–re-test reliabilities of the DPI. Inter-rater and test–re-test reliabilities (between sessions and within sessions) of the DPI for measuring pelvic tilt on the right and left sides, as assessed by intra-class correlation coefficient (ICC), standard error of measurement (SEM) and minimum difference (MD) to be considered real.

	Inter-rater		Test–re-test (between sessions)		Test–re-test (within sessions)	
	Right	Left	Right	Left	Right	Left
ICC	0.81^*^	0.88^*^	0.85^*^	0.65^*^	0.88^*^	0.95^*^
SEM	2.2	2.0	1.9	3.4	1.7	1.1
MD	6.0	5.5	5.4	9.4	4.8	2.9

**Notes.**

* = significant, *p* < 0.05.

Data for 18 subjects were available for inter-rater reliability and test–re-test reliability (within sessions) but data for only 16 subjects were available for test–re-test reliability (between sessions), as only 16 subjects attended both sessions. Subject attendance in each session, along with the raw data for the mean pelvic tilt on left and right sides is shown in Table 4.

**Table 4:** Raw data showing pelvic tilt. Raw data showing pelvic tilt for each session (mean of three individual measurements), for each rater, for each subject.

	Left side				Right side			
	Rater 1		Rater 2		Rater 1		Rater 2	
Subject	Session 1	Session 2	Session 1	Session 2	Session 1	Session 2	Session 1	Session 2
1	14.07	15.33	10.17	12.83	13.50	10.50	9.83	8.60
2	20.00	14.83	14.83	16.27	20.15	17.50	16.83	16.73
3	8.70	5.67	14.67	3.53	10.15	11.00	6.33	7.57
4	10.27	6.67	17.17	8.97	12.35	11.33	8.33	11.40
5	11.13	No data	22.33	No data	14.20	No data	17.83	No data
6	13.70	14.17	16.67	11.37	11.95	16.33	13.50	10.00
7	11.60	9.67	9.17	10.87	10.15	10.17	9.83	9.73
8	12.13	7.00	12.33	7.80	8.80	10.33	3.50	12.37
9	10.17	5.67	12.17	7.17	9.10	10.33	7.67	8.67
10	14.93	11.83	14.67	7.13	16.75	10.17	11.83	11.33
11	1.67	−3.50	0.67	−10.47	2.80	0.17	−3.00	−3.33
12	11.03	1.67	10.00	−4.47	10.10	5.67	8.00	2.30
13	15.17	13.50	15.17	12.33	15.20	11.83	12.83	12.93
14	7.80	6.67	9.00	5.73	6.45	10.00	3.33	1.43
15	16.77	16.17	18.17	11.57	17.25	20.50	9.67	15.60
16	16.37	10.33	14.33	9.23	17.30	15.33	10.67	14.20
16	10.43	7.33	8.33	6.40	10.95	8.33	5.50	9.47
18	No data	18.00	No data	12.13	No data	16.00	No data	13.80

## Discussion

The purpose of this study was to investigate the inter-rater reliability and test–re-test reliability of the DPI for measuring pelvic tilt angle on both right and left sides of the pelvis in young, healthy males and females. The first hypothesis for this study was that inter-rater reliability for the DPI would be good. The second hypothesis was that test–re-test reliability for the DPI would be good within a single rating session. The third hypothesis was that test–re-test reliability for the DPI would be good between two rating sessions.

By reference to pre-determined criteria for assessing reliability by reference to the magnitude of the ICC, the inter-rater reliability of the DPI for measuring pelvic tilt was designated as good on both sides (ICC = 0.81–0.88), the test–re-test reliability of the DPI for measuring pelvic tilt within a single rating session was designated as good on both sides (ICC = 0.88–0.95), and the test–re-test reliability for the DPI for measuring pelvic tilt between two rating sessions was designated as moderate on the left side (ICC = 0.65) and good on the right side (ICC = 0.85).

For inter-rater and test-rest reliability, our findings (ICC = 0.65–0.95; SEM = 1.9–3.4 degrees; MD = 2.9–9.4 degrees) are broadly in line with those of other investigations in similar devices measuring pelvic tilt. In their trial of a very similar type of caliper-based inclinometer to the DPI, Crowell et al. (1994) reported good intra-rater reliability (ICC = 0.92; SEM = 0.93 degrees; MD = 2.6 degrees) and good inter-rater reliability (ICC = 0.95; SEM = 0.78 degrees; MD = 2.2 degrees), Preece et al. (2008) reported good intra-rater reliability (albeit in cadavers) (ICC = 0.98; SEM = 1.1 degrees; MD = 3.1 degrees), Gnat et al. (2009) reported good intra-rater reliability (ICC = 0.99; SEM and MD not reported), Herrington (2011) reported good intra-rater reliability (ICC = 0.87; SEM = 1.1 degrees; MD = 2.5 degrees), and Fourchet et al. (2014) reported good inter-rater and intra-rater reliability (coefficient of variation = 15.8%). The reliability of the PALM in assessing linear differences in iliac crest height has also been found to be good (Hagins et al., 1998; Petrone et al., 2003) but whether such findings can be considered as directly comparable with the measurement of pelvic tilt angle is unclear. The reliability of a three-dimensional (3D) camera-based motion capture system reported by Levine & Whittle (1996) was also found to be good but interestingly no better than the PALM (ICC = 0.95; SEM = 0.96 degrees; MD = 2.7 degrees) and the caliper-based system used by Gajdosik et al. (1985) also displayed similar reliability (ICC = 0.88; SEM = 1.4 degrees; MD = 4.0 degrees).

Regarding pelvic tilt, our descriptive statistics (means of 10.5–10.6 degrees) are in line with the findings of other investigations, across various measurement devices. Using a PALM device, Herrington (2011) measured pelvic tilt in a population of 120 young, healthy subjects (65 males and 55 females, aged 23.8 years). It was reported that 85% of males and 75% of females displayed an anteriorly rotated pelvis, in the range of 6–7 degrees. Also using a PALM device, Lee, Yoo & Gak (2011) measured pelvic tilt in a population of 40 young, healthy subjects (23 males aged 23.8 years and 17 females aged 21.4 years) and found that anterior pelvic tilt was 7–8 degrees. Gajdosik et al. (1985) measured pelvic tilt in a population of 20 healthy males, aged 25.2 years, and reported a mean anterior pelvic tilt angle of 8.5 ± 4.1 degrees. Using a 3D camera-based motion capture system, Levine & Whittle (1996) measured pelvic tilt angle in a population of 20 healthy female subjects, aged 23.4 years, and reported a mean anterior pelvic tilt angle of 11.3 ± 4.3 degrees. Using radiography, Vaz et al. (2002) measured pelvic tilt angle in 100 healthy students from medical professions, aged 27 years, and reported a mean anterior pelvic tilt angle of 12.3 ± 5.9 degrees. From this very brief review, it seems that calliper or calliper-inclinometer systems (Gajdosik et al., 1985; Herrington, 2011; Lee, Yoo & Gak, 2011) tend to report slightly lower values of anterior pelvic tilt (6–8 degrees vs. 11–12 degrees) than those found using more sophisticated methods (Levine & Whittle, 1996; Vaz et al., 2002). It is interesting that the values reported here using the DPI (means of 10.5–10.6 degrees) are at the higher end of the spectrum reported in the literature and closer to those observed using more sophisticated methods. Whether this is a feature of the population measured, the presence of a spirit level in the DPI to standardize measurements relative to the ground, systematic bias in the DPI, or systematic bias in the raters is unclear.

Regarding differences between right and left sides, this investigation reported descriptive statistics (mean of 0.1 degrees greater anterior pelvic tilt on the right side) that are within the range of values observed by others. The literature is conflicting regarding whether the left or right sides tend to be more anteriorly rotated, or whether no difference is the norm. In respect of the prevailing direction of greater anterior tilt, some studies have reported very small differences that are likely within the bounds of measurement error (Gnat et al., 2009; Lee, Yoo & Gak, 2011). Other investigators have reported greater mean anterior tilt on the right side (Krawiec et al., 2003), which has been predicted based upon the apparent tendency for the right leg to be shorter in many populations (Knutson, 2005). However, greater mean anterior tilt on the left side has also been reported (Barakatt et al., 1996). In respect of the magnitude of difference between sides, as noted above, some studies have reported very small differences (Gnat et al., 2009; Lee, Yoo & Gak, 2011), while others have reported differences of around 2 degrees (Barakatt et al., 1996; Krawiec et al., 2003). It is noteworthy that Gnat et al. (2009) reported low mean values for the difference between sides in quiet standing (<0.5 degrees) but much greater values after exercise, particularly jumping (4.65 ± 1.56 degrees).

### Limitations

There are several key limitations to this investigation. The study design and consequently the forms of ICC used for statistical analysis do not permit the extrapolation of these results to any rater but rather limit their application to experienced and trained raters (Shrout & Fleiss, 1979). Different results might therefore be observed in untrained or in trained but inexperienced raters. In addition, the subjects who were assessed comprised young, healthy physical therapy students and investigations in other populations might yield differing findings. Care should therefore be taken in drawing inferences about the use of the DPI in the general population based on these results. There were also two key controls in which the study protocol was deficient. Firstly, the raters were not blinded to the values displayed on the DPI for each measurement, unlike some other studies assessing reliability in similar devices (Gnat et al., 2009). This limitation is of particular concern in relation to the test–re-test reliability measurement taken within a single session, where it was very easy for each rater to recall the previous measurement when taking additional measurements. Secondly, the activities of the subjects immediately prior to the measurements being taken were not controlled. Since mechanical loading has been found to affect pelvic tilt angle (Gnat & Saulicz, 2008; Gnat et al., 2009), this may have affected the reliability of the measurements taken between sessions. In addition, although our exclusion criteria prevented the inclusion of any subjects with medical conditions leading to clinically meaningful leg length discrepancies, our study was limited in that we did not perform any tests to assess whether any of the subjects had such leg length discrepancies, nor did we measure any other musculoskeletal parameters, such as hamstring and lumbopelvic flexibility using the sit-and-reach test, or actual hamstring muscle–tendon length. Such confounding factors might have affected our results.

In respect of the validity of the DPI, there are three substantial limitations of the present study. Firstly, criterion reference validity of the DPI for assessing anterior pelvic tilt on either side of the pelvis was not assessed. Future studies could explore this by correlating measurements taken using the DPI with measurements taken using gold standard methods (such as radiography) in the same group of subjects, as other investigators have done (Crowell et al., 1994; Petrone et al., 2003). Therefore, while the DPI displays good reliability between raters and between ratings taken in the same session, it may not produce valid measurements of pelvic tilt in comparison with values recorded using radiography or MRI. Secondly, the extent to which the measurements of anterior pelvic tilt on either side of the pelvis or the difference between these (pelvic torsion) might be predictive of increased injury risk or low back pain was not assessed. Thirdly, the extent to which measurements of anterior pelvic tilt on either side of the pelvis or the difference between these (pelvic torsion) might provide useful information about the extent of any existing leg length inequality was not explored.

## Conclusions

The inter-rater reliability and test–re-test reliability of the DPI for measuring pelvic tilt angle on both right and left sides of the pelvis were assessed, in a convenience sample of young, healthy males and females. The inter-rater reliability of the DPI for measuring pelvic tilt was designated as good on both sides (ICC = 0.81–0.88); the test–re-test reliability of the DPI for measuring pelvic tilt within a single rating session was designated as good on both sides (ICC = 0.88–0.95); and the test–re-test reliability for the DPI for measuring pelvic tilt between two rating sessions was designated as moderate on the left side (ICC = 0.65) and good on the right side (ICC = 0.85). Given that the raters were not blinded to the measurements, our findings regarding the test–re-test reliability of the DPI for measuring pelvic tilt within a single rating session should be interpreted with caution. Nevertheless, these results indicate that the DPI produces acceptably reliable measurements, although further research is required to establish the validity of the DPI in measuring pelvic tilt.

## Supplemental Information

10.7717/peerj.1881/supp-1 Supplemental Information 1 Data from first reliability trial

10.7717/peerj.1881/supp-2 Supplemental Information 2 Data from second reliability trial

## Additional Information and Declarations

### Data Availability

The following information was supplied regarding data availability: The raw data has been supplied as Supplemental Information.

## References

[ref-1] Alviso DJ, Dong GT, Lentell GL (1988). Intertester reliability for measuring pelvic tilt in standing. Physical Therapy.

[ref-2] Barakatt E, Smidt GL, Dawson JD, Wei SH, Heiss DG (1996). Interinnominate motion and symmetry: comparison between gymnasts and nongymnasts. Journal of Orthopaedic & Sports Physical Therapy.

[ref-3] Batterham AM, George KP (2003). Reliability in evidence-based clinical practice: a primer for allied health professionals?. Physical Therapy in Sport.

[ref-4] Betsch M, Wild M, Große B, Rapp W, Horstmann T (2012). The effect of simulating leg length inequality on spinal posture and pelvic position: a dynamic rasterstereographic analysis. European Spine Journal.

[ref-5] Blondel B, Parratte S, Tropiano P, Pauly V, Aubaniac JM, Argenson JN (2009). Pelvic tilt measurement before and after total hip arthroplasty. Orthopaedics & Traumatology: Surgery & Research.

[ref-6] Burdett RG, Brown KE, Fall MP (1986). Reliability and validity of four instruments for measuring lumbar spine and pelvic positions. Physical Therapy.

[ref-7] Chaléat-Valayer E, Mac-Thiong JM, Paquet J, Berthonnaud E, Siani F, Roussouly P (2011). Sagittal spino-pelvic alignment in chronic low back pain. European Spine Journal.

[ref-8] Clayson SJ, Newman IM, Debevec DF, Anger RW, Skowlund HV, Kottke F (1962). Evaluation of mobility of hip and lumbar vertebrae of normal young women. Archives of Physical Medicine and Rehabilitation.

[ref-9] Cooperstein R, Lew M (2009). The relationship between pelvic torsion and anatomical leg length inequality: a review of the literature. Journal of Chiropractic Medicine.

[ref-10] Crowell RD, Cummings GS, Walker JR, Tillman LJ (1994). Intratester and intertester reliability and validity of measures of innominate bone inclination. Journal of Orthopaedic & Sports Physical Therapy.

[ref-11] Cummings G, Scholz JP, Barnes K (1993). The effect of imposed leg length difference on pelvic bone symmetry. Spine.

[ref-12] Day JW, Schmidt GL, Lehmann T (1984). Effect of pelvic tilt on standing posture. Physical Therapy.

[ref-13] Flint MM (1963). Lumbar posture: a study of roentgenographic measurement and the influence of flexibility and strength. Research Quarterly. American Association for Health, Physical Education and Recreation.

[ref-14] Fourchet F, Materne O, Rajeb A, Horobeanu C, Farooq A (2014). Pelvic tilt: reliability of measuring the standing position and range of motion in adolescent athletes. British Journal of Sports Medicine.

[ref-15] Gajdosik R, Simpson R, Smith R, DonTigny RL (1985). Pelvic tilt intratester reliability of measuring the standing position and range of motion. Physical Therapy.

[ref-16] Gamer M, Lemon J, Fellows I, Singh P (2007). IRR: various coefficients of interrater reliability and agreement.

[ref-17] Gnat R, Saulicz E (2008). Induced static asymmetry of the pelvis is associated with functional asymmetry of the lumbo-pelvo-hip complex. Journal of Manipulative and Physiological Therapeutics.

[ref-18] Gnat R, Saulicz E, Biały M, Kłaptocz P (2009). Does pelvic asymmetry always mean pathology? Analysis of mechanical factors leading to the asymmetry. Journal of Human Kinetics.

[ref-19] Guenoun B, Zadegan F, Aim F, Hannouche D, Nizard R (2012). Reliability of a new method for lower-extremity measurements based on stereoradiographic three-dimensional reconstruction. Orthopaedics & Traumatology: Surgery & Research.

[ref-20] Gurney B (2002). Leg length discrepancy. Gait & Posture.

[ref-21] Hagins M, Brown M, Cook C, Gstalder K, Kam M, Kominer G, Strimbeck K (1998). Intratester and intertester reliability of the palpation meter (PALM) in measuring pelvic position. Journal of Manual & Manipulative Therapy.

[ref-22] Hallgren KA (2012). Computing inter-rater reliability for observational data: an overview and tutorial. Tutorials in Quantitative Methods for Psychology.

[ref-23] Heino JG, Godges JJ, Carter CL (1990). Relationship between hip extension range of motion and postural alignment. Journal of Orthopaedic & Sports Physical Therapy.

[ref-24] Herrington L (2011). Assessment of the degree of pelvic tilt within a normal asymptomatic population. Manual Therapy.

[ref-25] Juhl JH, Cremin TMI, Russell G (2004). Prevalence of frontal plane pelvic postural asymmetry—part 1. Journal of the American Osteopathic Association.

[ref-26] Knutson GA (2005). Anatomic and functional leg-length inequality: a review and recommendation for clinical decision-making. Part I, anatomic leg-length inequality: prevalence, magnitude, effects and clinical significance. Chiropractic & Manual Therapies.

[ref-27] Krawiec CJ, Denegar CR, Hertel J, Salvaterra GF, Buckley WE (2003). Static innominate asymmetry and leg length discrepancy in asymptomatic collegiate athletes. Manual Therapy.

[ref-28] Lalonde NM, Dansereau J, Pauget P, Cinquin P, Aissaoui R (2006). Accessing the influence of repositioning on the pelvis’ 3-D orientation in wheelchair users. IEEE Transactions on Neural Systems and Rehabilitation Engineering.

[ref-29] Lazennec JY, Rousseau MA, Rangel A, Gorin M, Belicourt C, Brusson A, Catonné Y (2011). Pelvis and total hip arthroplasty acetabular component orientations in sitting and standing positions: measurements reproductibility with EOS imaging system versus conventional radiographies. Orthopaedics & Traumatology, Surgery & Research.

[ref-30] Lee JH, Yoo WG, Gak HB (2011). The immediate effect of anterior pelvic tilt taping on pelvic inclination. Journal of Physical Therapy Science.

[ref-31] Levine D, Walker JR, Tillman LJ (1997). The effect of abdominal muscle strengthening on pelvic tilt and lumbar lordosis. Physiotherapy Theory and Practice.

[ref-32] Levine D, Whittle MW (1996). The effects of pelvic movement on lumbar lordosis in the standing position. Journal of Orthopaedic & Sports Physical Therapy.

[ref-33] Lim HS, Roh SY, Lee SM (2013). The relationship between pelvic tilt angle and disability associated with low back pain. Journal of Physical Therapy Science.

[ref-34] McGraw KO, Wong SP (1996). Forming inferences about some intraclass correlation coefficients. Psychological Methods.

[ref-35] Moes CCM (1998). Measuring the tilt of the pelvis. Ergonomics.

[ref-36] O’Sullivan P (2012). It’s time for change with the management of non-specific chronic low back pain. British Journal of Sports Medicine.

[ref-37] Petrone MR, Guinn J, Reddin A, Sutlive TG, Flynn TW, Garber MP (2003). The accuracy of the palpation meter (PALM) for measuring pelvic crest height difference and leg length discrepancy. Journal of Orthopaedic & Sports Physical Therapy.

[ref-38] Portney LG, Watkins MP (2008). Foundations of clinical research: applications to practice.

[ref-39] Preece SJ, Willan P, Nester CJ, Graham-Smith P, Herrington L, Bowker P (2008). Variation in pelvic morphology may prevent the identification of anterior pelvic tilt. Journal of Manual & Manipulative Therapy.

[ref-40] Sanders G, Stavrakas P (1981). A technique for measuring pelvic tilt. Physical Therapy.

[ref-41] Schache AG, Bennell KL, Blanch PD, Wrigley TV (1999). The coordinated movement of the lumbo–pelvic–hip complex during running: a literature review. Gait & Posture.

[ref-42] Schache AG, Blanch PD, Murphy AT (2000). Relation of anterior pelvic tilt during running to clinical and kinematic measures of hip extension. British Journal of Sports Medicine.

[ref-43] Schache AG, Blanch P, Rath D, Wrigley T, Bennell K (2002). Three-dimensional angular kinematics of the lumbar spine and pelvis during running. Human Movement Science.

[ref-44] Shrout PE, Fleiss JL (1979). Intraclass correlations: uses in assessing rater reliability. Psychological Bulletin.

[ref-45] Sprigle S, Flinn N, Wootten M, McCorry S (2003). Development and testing of a pelvic goniometer designed to measure pelvic tilt and hip flexion. Clinical Biomechanics.

[ref-46] Vaz G, Roussouly P, Berthonnaud E, Dimnet J (2002). Sagittal morphology and equilibrium of pelvis and spine. European Spine Journal.

[ref-47] Walker ML, Rothstein JM, Finucane SD, Lamb RL (1987). Relationships between lumbar lordosis, pelvic tilt, and abdominal muscle performance. Physical Therapy.

[ref-48] Walmsley RP, Amell TK (1996). The application and interpretation of intraclass correlations in the assessment of reliability in isokinetic dynamometry. Isokinetics and Exercise Science.

[ref-49] Weir JP (2005). Quantifying test–re-test reliability using the intraclass correlation coefficient and the SEM. The Journal of Strength & Conditioning Research.

[ref-50] Wild M, Kühlmann B, Stauffenberg A, Jungbluth P, Hakimi M, Rapp W, Betsch M (2014). Does age affect the response of pelvis and spine to simulated leg length discrepancies? A rasterstereographic pilot study. European Spine Journal.

[ref-51] Wolak ME, Fairbairn DJ, Paulsen YR (2012). Guidelines for estimating repeatability. Methods in Ecology and Evolution.

[ref-52] Wolak M (2012). Functions facilitating the estimation of the intraclass correlation coefficient.

[ref-53] Youdas JW, Garrett TR, Egan KS, Therneau TM (2000). Lumbar lordosis and pelvic inclination in adults with chronic low back pain. Physical Therapy.

[ref-54] Youdas JW, Garrett TR, Harmsen S, Suman VJ, Carey JR (1996). Lumbar lordosis and pelvic inclination of asymptomatic adults. Physical Therapy.

[ref-55] Young RS, Andrew PD, Cummings GS (2000). Effect of simulating leg length inequality on pelvic torsion and trunk mobility. Gait & Posture.

